# The Role of the Brain in the Pathogenesis and Physiology of Polycystic Ovary Syndrome (PCOS)

**DOI:** 10.3390/medsci7080084

**Published:** 2019-08-02

**Authors:** Eulalia A. Coutinho, Alexander S. Kauffman

**Affiliations:** Department of Obstetrics, Gynecology, and Reproductive Sciences, University of California, San Diego, 9500 Gilman Drive, La Jolla, CA 92093, USA

**Keywords:** PCOS, neuroendocrine, brain, LH, GnRH, GABA, Kisspeptin, Kiss1, androgen, pulses

## Abstract

Polycystic ovary syndrome (PCOS) is a common reproductive endocrine disorder, affecting at least 10% of women of reproductive age. PCOS is typically characterized by the presence of at least two of the three cardinal features of hyperandrogenemia (high circulating androgen levels), oligo- or anovulation, and cystic ovaries. Hyperandrogenemia increases the severity of the condition and is driven by increased luteinizing hormone (LH) pulse secretion from the pituitary. Indeed, PCOS women display both elevated mean LH levels, as well as an elevated frequency of LH pulsatile secretion. The abnormally high LH pulse frequency, reflective of a hyperactive gonadotropin-releasing hormone (GnRH) neural circuit, suggests a neuroendocrine basis to either the etiology or phenotype of PCOS. Several studies in preclinical animal models of PCOS have demonstrated alterations in GnRH neurons and their upstream afferent neuronal circuits. Some rodent PCOS models have demonstrated an increase in GnRH neuron activity that correlates with an increase in stimulatory GABAergic innervation and postsynaptic currents onto GnRH neurons. Additional studies have identified robust increases in hypothalamic levels of kisspeptin, another potent stimulator of GnRH neurons. This review outlines the different brain and neuroendocrine changes in the reproductive axis observed in PCOS animal models, discusses how they might contribute to either the etiology or adult phenotype of PCOS, and considers parallel findings in PCOS women.

## 1. Introduction

Polycystic ovary syndrome (PCOS) is a highly prevalent endocrine disorder and a leading cause of infertility, affecting at least 10% of reproductive-age women [1]. According to the Rotterdam criteria, for a diagnosis of PCOS, women have to exhibit two out of the three cardinal features: (1) oligo- or anovulation, (2) clinical and/or biochemical hyperandrogenism, and (3) polycystic ovaries [2]. In a large number of cases, PCOS is also frequently associated with a metabolic phenotype, including insulin resistance, increased adiposity, and cardiovascular diseases [3]. There is still no cure for PCOS, as the symptomology is complex and varied, and the etiology of the disease remains unknown. 

The reproductive abnormalities in PCOS women are thought to be the consequence of the high androgens and luteinizing hormone (LH) levels [4,5,6]. PCOS women not only have higher basal LH levels but also exhibit an increased number of LH pulses (i.e., pulse frequency) [4,7], together contributing to the abnormally high LH levels. This is of importance, because the elevated LH levels drive sex steroid (androgen and estrogen) synthesis by the ovarian theca cells and thus contribute to hyperandrogenemia in PCOS. Gonadotropin-releasing hormone (GnRH) neurons in the forebrain and preoptic area of the hypothalamus are the final node integrating all afferent signals to stimulate the pulsatile secretion of pituitary gonadotropins, LH and follicle-stimulating hormone (FSH) [8]. The pulsatile release of GnRH by hypothalamic GnRH neurons regulates LH and FSH levels [9]. Hence, abnormal LH secretion, especially in terms of altered LH pulse frequency, strongly points to hyperactive GnRH pulse secretion from the brain. 

The secretion of GnRH from the brain is itself regulated by a number of upstream neural and endocrine factors that contribute to both the timing and magnitude of GnRH secretion. Such regulators include hypothalamic populations of neurons expressing kisspeptin and gamma aminobutyric acid (GABA), amongst others. Whether any of these afferent regulators of GnRH neurons are themselves altered in PCOS remain unclear, but if so, may form the underlying basis for the hypersecretion of GnRH and LH in this disorder. Furthermore, direct action of androgens in the brain is thought to contribute to the manifestation of some PCOS traits [10]. This was demonstrated in a preclinical model of PCOS where neuron-specific deletion of androgen receptor (AR) prevented ovulatory dysfunction and reversed the increased adiposity induced by postnatal exposure to androgens [10]. Thus, increased androgen signaling in the brain may be a potential mechanism in the pathophysiology of PCOS. 

Over the last decade, there have been several studies providing genetic evidence to support the importance of abnormal gonadotropin secretion and signaling in hyperandrogenemia and PCOS traits. A recent study showed a strong association of LH and LHR gene polymorphisms with the occurrence of PCOS [11]. In addition, there have been genome-wide association studies (GWAS) that have identified variants in or near the LH/choriogonadotropin receptor (LHCGR) and FSH receptor (FSHR) genes, as well as a variant in the FSHβ (β-subunit of FSH) gene [12,13,14]. The latter gene variant results in the mutation of FSHβ, which causes FSH deficiency and is associated with high LH levels and impaired folliculogenesis in the ovary [15]. Thus, identification of susceptibility loci in these genes in PCOS women suggests that LH, LHCGR, FSHβ and FSHR genes may be involved in the etiology or pathology of PCOS. 

Several clinical studies have also implied a neuroendocrine basis to PCOS by demonstrating a positive correlation of PCOS occurrence and psychiatric disorders. In particular, there is a higher prevalence of PCOS in women with bipolar disorder (BPD) [16], thought to be associated with the effect of neurotransmitter imbalance on the neuroendocrine axis regulating reproduction. Similarly, there is a greater incidence of PCOS in women with epilepsy [17,18]. This is associated with transient increases in LH, FSH and prolactin levels [19], which is reflective of altered hypothalamic-pituitary function that might contribute to PCOS. Furthermore, there is a significant difference in the occurrence of PCOS in women treated with valproate, a drug used to treat epilepsy and BPD [20,21,22]. Valproate acts by increasing GABA levels and can alter neuron firing by blocking voltage-gated ion channels [23]. Hence, changes in GABA levels and action might be directly or indirectly modifying the hypothalamus-pituitary-gonadal (HPG) axis, a possibility discussed more in later sections. Taken together, these correlative relations also point to changes in the neuroendocrine axis as a possible contributing factor to the etiology of PCOS.

This review will highlight the insights gained into the neuroendocrine mechanisms underlying PCOS, focusing primarily on data gleaned from preclinical animal models of PCOS. First, we will briefly introduce the various PCOS animal models and summarize their neuroendocrine reproductive traits, focusing on gonadotropin and sex steroid levels. Following that, we will review recent findings on brain and neuroendocrine alterations at the cellular, molecular, and physiological levels, in various PCOS research models, and discuss how this may relate to the human PCOS condition.

## 2. Preclinical Models of PCOS

### 2.1. Need for Preclinical Animal Models

PCOS is a highly complex, multifactorial disorder with a range of both reproductive and, often, metabolic abnormalities. Due to its different phenotypes and range of manifestations, it is extremely challenging to identify the causes or underlying biological alterations of PCOS. In addition, limitations to clinical studies in terms of trial design, logistics, and ethics, create challenges for progress in identifying the etiology of PCOS. Furthermore, whereas ovarian and metabolic parameters are somewhat easier to assess, it is extremely challenging to investigate in vivo alterations in brain anatomy and function in humans, including PCOS women. Therefore, the establishment of preclinical animal research models has been highly beneficial in shedding light on potential causes and, in particular, neuroendocrine mechanisms underlying PCOS. Most of the PCOS animal models are based on the androgen excess and corresponding infertility seen in PCOS women and aim to mimic these parameters. While the various animal models may not absolutely represent true PCOS, the models recapitulate many of the phenotypes of the disease, allowing for investigators to identify possible underlying cellular, molecular, and physiological alterations that may be occurring in the actual PCOS disorder (Table 1). Thus, the preclinical models can be referred to as PCOS-like, but this does not diminish their value and merit for understanding and testing what may be happening mechanistically in PCOS women, with the hopes of identifying novel avenues for future therapeutics for the disorder.

### 2.2. Prenatally Androgenized Animals

One commonly studied hypothesis as to the origins of PCOS is fetal exposure to high androgen levels during prenatal development [24]. This hypothesis is built on reports showing that (1) women with PCOS have higher androgen levels during pregnancy compared to healthy pregnant women [25,26] and (2) there is a high occurrence of PCOS in daughters from PCOS women. These observations led to the development of prenatally androgenized (PNA) models of PCOS in which exposure of pregnant dams to exogenous testosterone (T) or dihydrotestosterone (DHT) during late gestation leads to a PCOS-like phenotype of the female offspring in adulthood. This PNA paradigm has been performed in several species, including rodents, sheep, and rhesus monkeys [27,28,29,30,31,32,33,34]. Female PNA rodents, sheep, and monkeys all exhibit hyperandrogenemia in adulthood. These animals also have other reproductive and some metabolic abnormalities seen in PCOS. In particular, adult PNA rats, generated with prenatal exposure to either T or DHT, have high mean LH levels and LH pulse frequency, along with impaired estradiol positive feedback [35,36]. Similarly, adult PNA mice, generated with prenatal DHT exposure, also display increased LH pulse secretion and impaired steroid hormone negative feedback [29]. These PNA mice also have disrupted estrous cycles, atretic ovarian follicles, and thecal cell hyperplasia [27,28,30]. Likewise, PNA monkeys prenatally exposed to testosterone propionate display polycystic ovaries and increased adiposity [32], and also have high LH levels and impaired negative feedback [31]. Finally, PNA sheep also develop PCOS-like reproductive and metabolic phenotypes in adulthood, with high mean LH levels and rapid LH pulse frequency, along with impaired steroid negative feedback [34,37].

### 2.3. Prenatal Treatment with AMH

In addition to prenatal treatment with exogenous androgens, a new PCOS mouse model has recently been developed utilizing prenatal exposure to exogenous anti-müllerian hormone (AMH) [38]. AMH, secreted from the ovaries, is increased in PCOS women and remains elevated during pregnancy [38]. Exogenous AMH has been demonstrated to stimulate GnRH neurons [39] and, hence, exposure to elevated AMH is thought to be able to drive enhanced reproductive hormone (GnRH and LH) secretion. Adult female mice that were exposed to elevated AMH in utero (PAMH mice) exhibit a reproductive phenotype similar to PNA mice, including increased LH pulse frequency, increased androgen levels, and disrupted estrous cyclicity [38]. In this model, the elevated AMH is hypothesized to drive increased GnRH neuron activity in the mother, which in turn, drives increased LH secretion and increased androgen synthesis, leading to a fetal environment with androgen excess (coming from the mother). Therefore, the PAMH mice display a similar phenotype to PNA mice, perhaps because they are both being exposed to elevated androgens during prenatal life. However, whether these two PCOS models are indeed working through the same cellular, molecular, and physiological pathways remains to be determined. 

### 2.4. Postnatally Androgenized Animals

Another preclinical PCOS model is generated by chronic exposure to androgens from the peripubertal period onward. Rodents, postnatally exposed to androgens, typically DHT, around the time of puberty, develop some features of a PCOS-like phenotype, though not as complete as the PNA models. Postnatal androgenized female rodents demonstrate metabolic symptoms, including increased adiposity and insulin resistance, and also display polycystic ovaries and disrupted estrous cycles [40,41]. However, in contrast to PNA animals, PAMH mice, and PCOS women, postnatally androgenized animals do not have high LH secretion; in fact, they have greatly reduced LH levels [42]. 

There are also postnatal rodent models treated with dehydroepiandrosterone (DHA/DHEA), although the findings in these models are conflicting, with regards to the reproductive phenotype and LH levels. In one study, mice postnatally treated with dehydroepiandrosterone (DHA/DHEA) displayed significantly elevated LH levels, however these mice did not display any reproductive phenotypes such as polycystic ovarian morphology or disrupted estrous cycles [40]. In another study in rats, postnatal DHA treatment disrupted estrous cycles and ovarian follicle development [43,44]. However, these DHA-treated rats displayed lower LH levels, in contrast to the PCOS condition in women. The main difference between these two DHA/DHEA studies, besides the species, was the duration of treatment. The study in mice implanted a pellet at PND 21 (prepuberty) for 90 days [40], whereas the rats were given DHEA either for 15 days from PND 21 (prepuberty) [44] or for 20 days from PND 55 (adults) [43]. Overall, the typically reported decreased LH levels in DHT and DHA postnatal models suggests that their neuroendocrine circuits are not altered similarly to that of PCOS women. Thus, these postnatal androgen models perhaps better represents an “androgen negative feedback” model rather than PCOS (at least from a neuroendocrine reproductive perspective).

### 2.5. Aromatase Inhibition Models

PCOS traits can also be induced by postnatal treatment with letrozole (LET), a non-steroidal aromatase enzyme inhibitor. Chronic LET treatment in rats from puberty to adulthood (5 or 10 weeks in total duration) disrupted estrous cycles, ovarian function, and increased both T and LH, mimicking the reproductive symptoms of PCOS [45,46]. Recently, another LET model was developed by administering LET to rats by oral gavage [47]. The treated rats had disrupted estrous cycles, arrested follicular development, and high T and LH levels. They also exhibited a strong metabolic phenotype, with increased body weight and glucose intolerance [47]. Similar to LET rats, chronic LET treatment in mice, initiated peri-pubertally for 5 weeks, induced both metabolic and reproductive abnormalities [48]. LET mice displayed disrupted estrous cycles, polycystic ovaries, and elevated LH and T levels, along with increased adiposity and glucose intolerance, thereby recapitulating both the metabolic and reproductive traits observed in many PCOS women [48]. In addition to the elevated T and LH levels, there was also a decrease in FSH [48]. It is unclear if the elevated LH levels are due to impaired negative feedback owing to reduced estradiol (E2) or heightened androgen action or both. These findings are consistent with elevated T and LH levels in some PCOS women with low E2 levels. Due to the elevated LH levels and strong reproductive phenotype in all LET models, these models are useful to study possible changes in reproductive neural circuits in PCOS-like conditions. 

In general, the LET models in rats and mice tend to induce an overweight or obese PCOS-like phenotype, in contrast to the PNA model which has weaker or absent metabolic phenotype. Thus, the LET model may nicely represent a PCOS condition with a corresponding metabolic dysfunction, whereas the PNA model may better represent the lean PCOS condition. This is important, as PCOS women exhibit a range of features and not all PCOS cases may be caused by the same underlying alterations. Thus, having different animal models for different PCOS phenotypes may be informative to providing mechanistic insight into each unique PCOS “subtype”. It also means that the changes and impairments identified in the various PCOS models need not be the same, and discrepancies between models may in fact be related to different PCOS subtype etiologies or adult phenotypes.

## 3. Cellular and Molecular Mechanisms Contributing to the PCOS Neuroendocrine Phenotype

### 3.1. Implications of GnRH and LH Pulse Frequency vs. Amplitude

As stated earlier, the androgen excess observed in PCOS is thought to be a downstream result of the increased LH levels. Many clinical studies have reported an increase in both the frequency and amplitude of LH secretion in PCOS women [4,5,6]. As detailed in Section 2, and, consistent with the clinical findings, there is also an increase in mean LH levels in PNA [30,31,32,35,37,42,49,50], PAMH [38] and LET [45,46,48,51] models of PCOS. Some of the studies in PNA and PAMH models have gone beyond single, one-off measures of LH to assess repeated LH measures over time, identifying clear increases in LH pulse frequency [30,35,36,38] and LH pulse amplitude [37]. However, this information is missing in the postnatally treated DHT and LET models, and studies performing multiple blood sampling over several hours in these models are needed to assess possible changes in LH pulse frequency and amplitude. Nevertheless, the overall high LH levels seen in PCOS women and most preclinical PCOS animal models point to an abnormal hyper-activated hypothalamic-pituitary function. Because the amplitude of LH pulses can be regulated at both the level of the brain (GnRH output) or pituitary gonadotrope, alterations in basal LH levels or LH pulse amplitude may implicate changes occurring at either tissue. However, frequency of LH pulses are entirely due to the timing of GnRH pulse secretion, which itself may be dictated by upstream “GnRH pulse generator” alterations [52] or by intrinsic changes in GnRH neurons themselves. The fact that PCOS women exhibit alterations in LH pulse frequency directly implicates upstream changes in GnRH pulse frequency and its neural network. In the next sections, we will highlight evidence supporting the likelihood of changes in the brain, specifically in GnRH neurons and afferent hypothalamic GABA and/or kisspeptin neurons, in contributing to the neuroendocrine mechanisms underlying PCOS.

### 3.2. GnRH Neurons

The pulsatile release of GnRH at the median eminence controls the frequency of LH pulse secretion by the anterior pituitary. In addition to controlling the timing of LH secretion (i.e., pulse frequency), GnRH also modulates gonadotropin subunit mRNA expression and LH synthesis [53,54,55], thereby contributing to the absolute amount of LH released at any given time (i.e., basal levels and also, pulse amplitude). Women with PCOS have high LH pulse frequency and amplitude [4,7]. The latter implicates changes in either the pituitary or the brain, or both. In contrast, the former reflects an increase specifically in the brain, either in GnRH neurons themselves or upstream controllers of GnRH secretion. 

In line with the hypothesis for altered brain function, studies in PNA female mice have shown that GnRH neuron action potential firing activity is increased compared to that in control females [56]. In addition, there is an increase in both frequency and size of GABAergic postsynaptic currents (PSCs) to GnRH neurons in PNA females, suggesting an enhanced GABAergic drive to GnRH neurons. In congruence, another study demonstrated an increase in GABAergic synapses on GnRH neurons in PNA mice [30]. Similarly, in female PAMH mice, there was an increase in GnRH neuron firing and this correlated with an increase in number of GABA inputs onto GnRH neurons [38]. These findings, taken together along with GABA being excitatory to GnRH neurons through activation of GABA_A_ receptors, suggest that GnRH neurons’ hyperactivity drives the increased LH secretion seen in these PCOS-like models [57]. 

Besides the increase in excitatory GABAergic signals to the GnRH neurons, a decrease in inhibitory steroid hormone feedback is also a suggested cause for increased GnRH activity. To support this, it has been shown that PCOS women need a higher dose of estradiol and progesterone to achieve a similar reduction in mean LH levels, LH pulse frequency, and LH pulse amplitude as control healthy women [58]. There is also compelling evidence of impaired steroid hormone feedback in the PNA PCOS model [29,30]. PNA mice displayed a blunted LH response after ovariectomy and estradiol (E2) treatment, implicating impaired E2 negative feedback control of GnRH/LH secretion in PNA mice. Since GnRH neurons only express estrogen receptor (ER) β [59] and not the classical ERα [60] or androgen receptor (AR) [61], which are the main receptors mediating negative feedback, the impaired steroid feedback is likely mediated by afferent neurons. Thus, alterations in afferent neurons in the GnRH neural circuit that express steroid hormone receptors may lead to the hyperactivity of GnRH neurons. This will be discussed more in Section 3.3 and Section 3.4.

### 3.3. GABAergic Neurons

An important neuron population in the GnRH neural circuit are GABA cells in the medial basal hypothalamus. In the forebrain and many other areas of the brain, GABA is the main inhibitory neurotransmitter. However, due to the high intracellular chloride concentration in GnRH neurons, GABA actually *excites* these neurons rather than inhibits them. GABA’s stimulation of GnRH neurons occurs through the GABA_A_ receptor [57]. 

Recent findings have shown that levels of GABA are increased in the cerebrospinal fluid of PCOS women [62]. Given GABA’s stimulatory actions on GnRH neurons, these elevated CSF levels of GABA might contribute to the enhanced GnRH secretion in PCOS women, although this remains speculation at present. More convincingly, in studies in the PNA and PAMH mouse, GABA neuron axon inputs to the GnRH neurons were found to be increased [30,38]. Furthermore, the increased GABA fiber inputs originated specifically from cells in the arcuate nucleus (ARN) [30]. As already noted above, studies in PNA mice also reported an increase in GABAergic postsynaptic currents onto the GnRH neurons [28]. These findings highlight a possible role of GABA neurons in the hyperactivity of GnRH neurons in PCOS, at least in the PNA and PAMH models. 

In addition to changes in GABA signaling to the GnRH neurons, the regulation of GABA neurons themselves is also altered in PCOS animal models. The ARN GABA neurons of PNA females exhibit a decrease in expression of progesterone receptor (PR), perhaps contributing to the impaired steroid hormone feedback seen in this PCOS model [29,30] and matching the decreased progesterone feedback phenotype in some PCOS women [63]. The impaired progesterone feedback in PCOS women was also demonstrated in a study where PCOS women needed higher levels of progesterone to decrease LH compared to healthy women [58]. In the LET mouse model of PCOS, there is also a decrease in *PR* mRNA expression in the ARN region, as quantified by qPCR in mediobasal hypothalamic punches [48]. Further studies are needed to assess the specific neuron types that have decreased PR expression in the ARN region and determine if it is also the GABA neurons, as in the PNA mice; nonetheless, the diminished PR levels in LET mice suggest the possibility of diminished progesterone feedback in this model as well. Thus, GABA neurons, specifically ARN GABA neurons, might be a critical population to mediate normal progesterone negative feedback to the GnRH system, which is altered in a PCOS-like condition. 

Surprisingly, apart from the PNA and PAMH mice, there are almost no reports of ARN GABA levels, number of GABA neurons, or GABA signaling to GnRH neurons in other animal models of PCOS. Recently, in a different LET model also exhibiting high LH levels, where rats received LET by oral gavage, there was a decrease in GABA mRNA levels in several brain regions, including the hypothalamus [47]. In this same LET model, co-administration of GABA with LET decreased body weight and T levels compared to that of animals given just LET [64]. These findings would suggest that GABA is actually inhibitory to the reproductive axis, and that PCOS-condition has diminished inhibitory GABA signaling. This contrasts the stimulatory GABA story in the PNA mouse model, but the two findings are not necessarily in conflict, as GABA can act on multiple neuron populations in the brain and can be stimulatory in some (GnRH neurons) and inhibitory in others. The decrease is GABA levels could lead to increased activity of afferent neurons such as kisspeptin neurons, which are stimulatory to GnRH neurons. However, this is just speculation and further investigation is needed to test this hypothesis. Thus, where GABA is acting—or not acting—in most of the in vivo studies is not always clear and remains to be determined.

### 3.4. Kisspeptin Neurons

Kisspeptin, encoded by the *Kiss1* gene, is a 54-amino-acid protein that acts through the membrane receptor Kiss1r [65]. Kisspeptin is a potent stimulator of GnRH neurons [66]. In PCOS women, there is a positive correlation of circulating kisspeptin levels in the blood with the high circulating LH levels, though the source of this circulating kisspeptin (brain, liver, pancreas, adipose, gonad, etc.) is not immediately clear [67,68]. These findings, along with data from kisspeptin or Kiss1r knockout studies demonstrating an indispensable role of kisspeptin and Kiss1r in GnRH/LH secretion [69,70], suggest that kisspeptin-Kiss1r signaling may be a good candidate for an altered neural mechanism in the PCOS condition.

Kisspeptin is expressed in several tissues, including the brain and several peripheral tissues, but studies have determined that neural kisspeptin signaling specifically to GnRH neurons is the primary mode by which kisspeptin stimulates the reproductive axis [71,72]. Within the brain, kisspeptin is primarily expressed in the hypothalamic arcuate nucleus (ARN) and the anteroventral periventricular nucleus and periventricular nucleus continuum (AVPV/PeN), regions implicated in the regulation of GnRH secretion [65,73]. Lower degrees of kisspeptin expression occur in the medial amygdala [74], bed nucleus of the solitary tract (BNST), and lateral septum [75], but the roles of these extra-hypothalamic kisspeptin neurons remains unknown. The two hypothalamic kisspeptin populations are thought to serve different roles, with the ARN cells being involved in stimulating GnRH/LH pulses in both females and males [52], while the AVPV/PeN cells regulate the preovulatory GnRH/LH surge in females [65]. ARN kisspeptin neurons are unique in terms that they co-express Neurokinin B (NKB) and Dynorphin (Dyn), and hence, are known as the KNDy neurons [76]. Both NKB and Dyn play a role in modulating kisspeptin neuron activity and downstream regulation of GnRH/LH secretion [77]. NKB, encoded by the *TAC3* gene in humans and *Tac2* gene in rodents, acts via the NKB receptor (NK3R; encoded by *TACR3* gene in humans and *Tacr3* in rodents) [78], and mutations in either *TAC3* or *TACR3* are associated with hypogonadotropic hypogonadism [79]. Dyn acts on the kappa opioid receptor to decrease LH secretion [80], likely mediated via inhibition of KNDy neuron activity. In non-human primates, kisspeptin and NKB-NK3R signaling have been shown to increase GnRH release [81]. 

ARN KNDy neurons are negatively regulated by sex steroids, via androgen and estrogen receptor pathways [82,83], and are likely responsible for driving the hyperactive GnRH secretion pattern after removal of sex steroids via gonadectomy. On the other hand, AVPV/PeN kisspeptin neurons are positively regulated by elevated estradiol [82,83] and progesterone [84] levels leading up to the time of ovulation. In humans, kisspeptin neurons are present in the infundibular nucleus of the hypothalamus of both sexes [85], and in the rostral periventricular zone of females only [86]. These periventricular zone kisspeptin neurons may be analogous to the AVPV/PeN kisspeptin neurons seen in rodents known to mediate positive steroid feedback. Because PCOS women display abnormal increases in LH pulse secretion, most PCOS animal studies have focused on the ARN kisspeptin neurons, homologous to the human infundibular kisspeptin neurons.

In PNA female rats, there is a significant increase in the number of ARN kisspeptin- and NKB positive cells, as measured with immunohistochemistry, compared to control females [42]. Consistently, another study in PNA rats reported increased *Kiss1* and *Tac2* (the gene for NKB) mRNA expression in the ARN [36]. In PNA sheep, there is no increase in the number of kisspeptin cells; however, there is an increase in the size of kisspeptin neurons [87]. Because kisspeptin normally activates GnRH neurons, the observed increase in the number of kisspeptin cells or *Kiss1* mRNA could be a potential cause of increased GnRH neuron activity and LH secretion seen in this PNA model of PCOS. 

In postnatally treated models of PCOS, the effect on kisspeptin neurons has been investigated in animals treated with DHT or LET. In rats postnatally treated with DHT, there was a decrease in *Kiss1* mRNA in the hypothalamus, as well as a decrease in kisspeptin-ir cells in the ARN [88]. This is congruent with a decrease in LH compared to controls in this model, rather than an increase in LH as observed in PCOS [42]. On the other hand, in the rat LET PCOS model, in which females show high LH levels, there is a corresponding increase in the number of ARN kisspeptin cells compared to control females [89].

Overall, the number of kisspeptin cells and the amount of *Kiss1* and *Tac2* mRNA in the ARN positively correlate with LH levels in rodent PCOS models. Since kisspeptin stimulates GnRH neurons and NKB stimulates kisspeptin neurons via the NK3 receptor [90], the findings in the PCOS models suggest a role of the ARN KNDy neurons in driving GnRH hyperactivity and downstream elevated LH pulse secretion. In support of this, a clinical trial in PCOS women recently attempted to target KNDy neurons and decrease their activity to reduce LH secretion. This study treated PCOS women with an NK3 receptor antagonist [91] for 28 days and found that the treatment significantly reduced LH pulsatility and, subsequently, serum T concentrations. This was a phase 2 clinical trial and, therefore, did not evaluate long-term, post-treatment serum LH or T levels or ovulation; nonetheless, it demonstrated that the kisspeptin-NKB system is a promising therapeutic target to lower LH and T in PCOS women.

### 3.5. Contributions and Effects of Androgen Signaling in the Brain

Given the typically prevalent high circulating androgen levels in PCOS women, it is essential to dissect the contribution of such hyperandrogenemia to the pathophysiology of PCOS. An outstanding debate is whether the elevated androgens in PCOS women are merely a downstream endocrine response to hyperactive GnRH and LH secretion driving the ovary, or do the elevated androgens themselves act in the brain (or pituitary) during development and/or adulthood to sculpt and maintain the hypersecretion of GnRH and LH? Indeed, the mechanisms by which elevated androgens may mediate the changes in the neuroendocrine axis still remain unclear, and deciphering neural targets of androgen action may provide important information for development of treatments for PCOS. In PCOS women, treatment with flutamide decreased LH levels after E2 and P administration similar to that of control women [92]. This is of particular importance, because the same E2 and P administration failed to lower LH in PCOS women with no flutamide treatment [58], suggesting that hyperandrogenemia contributes to impaired steroid (E2 or P) hormone feedback in PCOS. Consistent with these findings, a recent study in the PNA PCOS model has shown that treatment with flutamide, an androgen receptor antagonist, can reverse abnormal GnRH neuron morphology, decrease T levels, and restore estrous cyclicity in PNA mice [93], suggesting that hyperandrogenemia is causing, directly or indirectly, some of the GnRH abnormalities (other aspects of GnRH physiology or upstream afferent circuits were not studied). This finding also highlights the promise of anti-androgenic drugs in the treatment of PCOS. However, since this study administered flutamide peripherally, it is not clear whether the androgen blockade occurring directly in the brain or other tissues was responsible for restoring these reproductive parameters, and if it was in the brain, in what specific cell targets? In support of the importance of androgen signaling specifically in the brain, a recent study in postnatally androgenized mice showed that androgen receptor (AR) knockout in the brain (using Cre/lox technology) improved ovarian morphology and function and decreased adiposity [10]. In that study, AR was assumed to be deleted in all neurons, and thus the exact brain region(s) and specific neuron types involved still remain to be elucidated. Nonetheless, these studies demonstrate that androgen actions in the brain may play a role in the manifestations of PCOS and that androgen-induced alterations in the GnRH neural network are likely to be important in the pathogenesis of many cases of PCOS. 

One major caveat to the theory that androgens are inducing hyper GnRH secretion is that androgens typically have been shown to provide negative feedback on the HPG axis, including in females. Thus, one would expect high androgen levels to provide heightened negative feedback and lower GnRH/LH secretion, rather than reducing negative feedback and facilitating increased GnRH/LH secretion. Indeed, androgen treatments not only reduce LH secretion in vivo, but also lower ARN *Kiss1* levels [82,94], further driving decreased GnRH output. This discrepancy with known negative feedback actions of androgen actions needs to be resolved in future studies postulating a role for androgens in actually promoting the hypersecretion of GnRH in PCOS.

## 4. Concluding Remarks and Perspectives

Despite its high prevalence, there is still no cure for PCOS. Clinical studies in the past few decades have significantly contributed to characterization of the condition and have also shed light on the different phenotypes and its occurrence. However, due to challenges associated with logistics and ethical issues, there has been limited progress in identifying the root causes of PCOS, especially at the level of the brain and pituitary. Development of preclinical animal models has been extremely helpful in addressing the hypotheses around the neuroendocrine contributions to PCOS. These animal models have established an important role of the brain in the possible pathogenesis of PCOS. Studies in PNA and PAMH mice have clearly demonstrated the presence of a hyperactive GnRH system that drives the increased LH levels in these PCOS-like conditions [29,30,38]. LET models of PCOS also nicely show elevated mean LH levels in both rats and mice, although specific demonstration of increased frequency of LH pulses in these LET models has yet to be reported.

**Figure 1:**
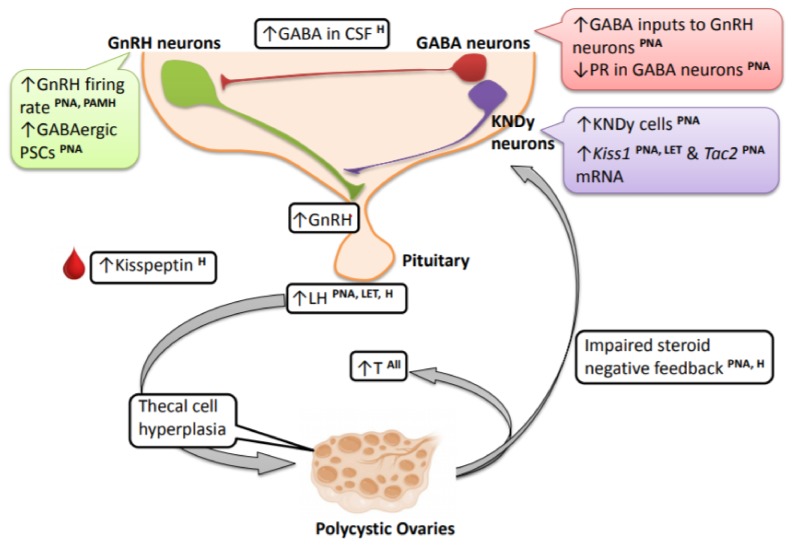
Changes in the brain and neuroendocrine reproductive axis in PCOS women and PCOS-like animal models. Clinical studies demonstrate very high circulating LH levels in PCOS individuals, and this is recapitulated in the prenatally androgenized (PNA) and letrozole (LET) preclinical animal models. These high-frequency LH pulses contribute to thecal cell hyperplasia and stimulate increased production and secretion of ovarian androgens, contributing to hyperandrogenemia. The elevated LH levels and LH pulse frequency seen in PCOS and the PCOS animal models point to an upstream hyperactive GnRH neural circuit. In PNA mice and mice prenatally treated with AMH (PAMH), there is an increased firing rate of GnRH neurons. PNA mice also exhibit an increase in upstream GABAergic postsynaptic currents (PSCs). There is also impaired sex steroid negative feedback observed in PNA models and PCOS women, and this may be mediated by alterations in afferent hypothalamic GABA neurons, at least in PNA mice. In relation to this, progesterone receptor (PR) expression is decreased in ARN GABA neurons in PNA mice and in the mediobasal hypothalamus of LET mice. Alongside GABA, there is also an increase in ARN KNDy cells and ARN *Kiss1* and *Tac2* mRNA levels in PNA and LET PCOS models, possibly providing an increase in excitatory signals to GnRH neurons. Refer to the article text for discussion on species differences and specific brain parameters that have not yet been assessed in different models. [LH—luteinizing hormone; PNA—prenatally androgenized animals; LET—letrozole-treated PCOS models; H—human/clinical data from PCOS women].

This review has also touched on the possible contribution of afferent neurons, kisspeptin and GABA neurons, to the hyperactivity of the GnRH neurons (Figure 1). However, it is to be noted that there are still missing pieces of information in some models with regard to GnRH neurons and its afferent neural network. Nonetheless, alongside clinical studies showing increased CSF GABA [62] and blood kisspeptin [67,68] levels in PCOS women, and an improvement in T and LH levels after treatment with a NK3R antagonist in PCOS women [91], the involvement of the neuroendocrine axis in the pathogenesis of PCOS is becoming more convincing. However, there are still a number of important unanswered questions that need addressing. Identifying specific neuron populations and signaling factors altered in PCOS is key to the development of potential therapeutics. Besides KNDy and GABA neurons, there may be other potential neurons involved in directly or indirectly modulating GnRH neuron function that may be affected in PCOS. Since PCOS is often also a metabolic disorder, with increased adiposity and impaired insulin sensitivity, it is likely that metabolic-sensitive neurons (e.g., AgRP/NPY and POMC neurons) may also contribute to the neuroendocrine mechanisms underlying PCOS. Thus, future studies investigating the contribution of other neuron populations and the neuropeptides and neurotransmitters they release are needed to get a complete picture of the neuroendocrine origins and adult phenotype of PCOS. In addition, identifying developmental time periods during which these neurons and circuits are altered will provide important information about “critical periods” for therapeutic targeting of these circuits.

## Figures and Tables

**Table 1:** Characteristics of polycystic ovary syndrome (PCOS) and alterations in the neuroendocrine axis in preclinical PCOS animal models.

Parameter	Prenatally Androgenized Animals (PNA)	Postnatally Androgenized Animals	Letrozole Treated Animals	Clinical Data
Hyperandrogenemia	Mouse—Yes	Mouse, Rat—High dihydrotestosterone (DHT) due to exogenous DHT treatment, but no increase in testosterone (T)	Mouse—Yes	Yes (60% according to the Rotterdam criteria)
	Rat—Yes		Rat—Yes	
	Sheep—Yes	-	-	
	Monkey—Yes	-	-	
Luteinizing hormone (LH) levels	Mouse—High + ↑pulse frequency	Mouse—No change	Mouse—High	High LH pulse frequency and amplitude
	Rat—High + ↑pulse frequency	Rat—No data	Rat—High	
	Sheep—↑↓ (Conflicting reports)	-	-	-
	Monkey—High	-	-	-
Negative feedback	Mouse—Impaired	Mouse—No data	Mouse—No data	Need higher doses of Estradiol and progesterone to decrease LH to similar levels in healthy women
	Rat—No data	Rat—No data	Rat—No data	
	Sheep—Impaired	-	-	
	Monkey—Impaired	-	-	
GnRH/GnRH neurons	Mouse (PNA)—↑GnRH firing rate; ↑GABAergic inputs to GnRH neurons; ↑GABAergic postsynaptic currents Mouse (PAMH)—↑GnRH firing rate; ↑GABAergic inputs to GnRH neurons	Mouse—No data	Mouse—No data	No data
	Rat—No data	Rat—No data	Rat—No data	
	Sheep—No data	-	-	
	Monkey—No data	-	-	
Kisspeptin/Kisspeptin neurons	Mouse—No data	Mouse—No data	Mouse—No data	Positive correlation between kisspeptin and LH levels; NK3R antagonist treatment decreased LH and T levels in women with PCOS
	Rat—↑Kiss and NKB positive cells in ARN; ↑*Kiss1* and *Tac2* mRNA levels	Rat—↓*Kiss1* mRNA in the hypothalamus ↓Kiss-ir cells in ARN	Rat—↑ARN Kiss cells	
	Sheep—No change Kiss cells, but ↑ in cell size of ARN Kiss cells; ↓excitatory glutamatergic inputs to Kiss cells	-	-	
	Monkey—No data	-	-	
GABA/GABA neurons	Mouse—↑GABAergic inputs from ARN to GnRH neurons; ↓Progesterone receptor in ARN GABA neurons	Mouse—No data	Mouse—No data	Increased GABA in CSF of PCOS women
	Rat—No data	Rat—No data	Rat (LET by oral gavage)—↓GABA mRNA levels in several brain regions including the hypothalamus; Co-administration of GABA with LET decreased T levels and body weight	
	Sheep—No data	-	-	
	Monkey—No data	-	-	
